# Muscle responses to limb block during spontaneous movements in infants

**DOI:** 10.3389/fnins.2025.1657677

**Published:** 2025-11-25

**Authors:** Damiana Rubeca, Irina Y. Dolinskaya, Victor A. Selionov, Elena S. Keshishian, Irina A. Solopova, Francesca Sylos-Labini, Francesco Lacquaniti, Yury Ivanenko

**Affiliations:** 1 Laboratory of Neuromotor Physiology, IRCCS Fondazione Santa Lucia, Rome, Italy; 2 Department of Systems Medicine and Center of Space Biomedicine, University of Rome Tor Vergata, Rome, Italy; 3 Laboratory of Neurobiology of Motor Control, Institute for Information Transmission Problems, Moscow, Russia; 4 Department of Neonatology, Moscow Research Institute of Clinical Pediatrics of Russian Federation, Moscow, Russia

**Keywords:** spontaneous movements, limb block, muscle responses, preterm infants, early development

## Abstract

Young infants manifest prominent neuromuscular responses to changes in muscle length, along with a variety of spontaneous movements. The first months of life are an important period during which sensorimotor integration and muscle tone gradually mature. In adults, muscle responses may also be observed when coordinated limb movements are transiently blocked. Given that infants normally exhibit spontaneous limb activity, here we examined whether a transient upper or lower limb block evoked consistent muscle responses while the infants were allowed to perform spontaneous movements with the other limbs. We examined polymyographic recordings in 12 bilateral arm and leg muscles in full-term and preterm infants (1–7 months old). Overall, muscle activity, its spectral characteristics, and agonist–antagonist coactivation were comparable before and after the block in both full-term and preterm infants, suggesting that the transient limb immobilization effect is not evident or consistent, as opposed to previously reported prominent muscle responses to muscle lengthening or shortening. The lack of consistent muscular responses to limb block supports the idea that individual limb motion during spontaneous movements is relatively independent of the control of other limbs, and that sensory input during changes in muscle length is more effective in revealing sensorimotor connections than its non-appearance.

## Introduction

Researchers interested in developmental biology and clinical perspectives may be particularly interested in examining how infants’ muscles react to active and passive movements or sensory stimulation during early development (Andrews and Fitzgerald, 1999; Cornelissen et al., 2013; Solopova et al., 2019; Dolinskaya et al., 2023). Kinematic characteristics and inter-limb coordination are being frequently used to assess general movements (Thelen et al., 1987; Piek and Carman, 1994; Vaal et al., 2000; Gima et al., 2011; Karch et al., 2012; Ohmura et al., 2016; Zuzarte et al., 2019), and alterations of kinematic patterns or the lack of variations in the way spontaneous movements (SMs) occur may be signs of early impairments (Prechtl et al., 1997; Kwong et al., 2018). Nonetheless, the examination of muscle activity offers further information on the maturation of the neuromuscular spinal and supraspinal control despite high inter-trial variability in infants (Ritterband-Rosenbaum et al., 2017; Sylos-Labini et al., 2022; Kanazawa et al., 2023). This includes the irradiation of sensory responses to distant or antagonist muscles, as well as the manifestation of prominent muscle responses to muscle lengthening and shortening in young infants (Myklebust and Gottlieb, 1993; Teulier et al., 2011; Solopova et al., 2019).

The way muscles react to changes in muscle length can be interpreted as signs of dynamic muscular tone associated with postural resistive or compliant behaviour (Cacciatore et al., 2024). Muscle responses can be seen not only in lengthening and shortening, but also in limb immobilization, especially if it is imposed during ongoing movements of the upper and lower limbs. For instance, in adults, they may be observed when coordinated limb movements are transiently blocked, causing consistent aftereffects, such as the continuation of rhythmic muscle activity imposed by preceding limb movement or the appearance of tonic postural activity (Gurfinkel and Ivanenko, 1987; Sylos-Labini et al., 2014). In neonates, a transient lower limb block during stepping movements may significantly affect ipsilateral proximal muscle activities, while the sudden release of the blocked limb may elicit the immediate initiation of the swing phase, with hip flexion and a burst of an ankle flexor muscle (Dewolf et al., 2022). Therefore, if limb block is viewed as a disruption to continuing coordinated inter- or intra-limb activity, it could be used as a probe to determine the presence or the level of inter-limb coordination or interactions between tonic and phasic muscle activity.

Here, we examined muscle responses to the transient limb block during active wakefulness in infants (1–7 months old) while testing supine and making spontaneous limb movements, which is typical for this age group. Previous research has also shown that preterm infants may have higher rates of muscular responses and a different time course of muscle power development (Cioni and Prechtl, 1990; de Groot et al., 1992; Dolinskaya et al., 2023), therefore we included both full-term and preterm infants in this investigation. Manual manipulations of flexion and extension joint movements are commonly used to assess muscle tone in infants in clinical settings (McIntyre et al., 2011; Marinelli et al., 2013), and we also used a manual limb block done by the same experimenter. We aimed at investigating the presence of consistent muscle responses to either blocking a single upper or lower limb or blocking both upper and lower limbs at once, while the infants were free to continue spontaneous movements with other limbs. To this end, we analysed electromyographic (EMG) recordings from 12 bilateral arm and leg muscles, as well as changes in the level of muscle activity, spectral characteristics, and against-antagonist coactivation before and after the block. The rationale of using these analyses is that they have been demonstrated to be successful in detecting changes in muscle activity characteristics during stepping and kicking movements in infants during their first year of life (Teulier et al., 2012; Sylos-Labini et al., 2020, 2022).

## Methods

### Participants

Participants were 20 full-term infants (7 females and 13 males, from 0.5 to 7.5 months postnatal age) and 18 preterm infants (10 females and 8 males, from 1 to 7 months corrected age at the time of investigation) (Table 1). For full-term infants, inclusion criteria were: an Apgar score >7 at 1 and 5 min, no delivery events or perinatal history, no known neurological or musculoskeletal issues, and a gestational age (GA) > 38 weeks. For preterm infants, criteria included an Apgar score >7 at 1 and 5 min, clinical stability at measurement, and a birth GA > 25 weeks. The exclusion criteria were congenital malformations or infections, genetic and metabolic diseases, malignant disorders, and ongoing mechanical ventilation therapy. Overall, the characteristics of the preterm infants were: birth at 25–36 weeks of GA and birth weight 0.6–3.2 kg. Two of them were extremely preterm (25–28 weeks of GA), one was very preterm (29–31 weeks of GA), seven were moderately preterm (32–33 weeks of GA), and eight were late preterm (34–36 weeks of GA) (Table 1). Experiments were performed at the Moscow Research Institute of Clinical Pediatrics. The study was carried out in accordance with the Declaration of Helsinki for experiments on humans, and the protocol had been approved by the Ethics Committee of the Moscow Research Institute of Clinical Pediatrics (protocol n.14/18). The infant’s parent gave informed written consent to participate in the study.

**Table 1:** Characteristics of full-term and preterm infants, recorded blocked limb conditions (“arm,” “leg,” “ipsi arm-leg,” “contra arm-leg”) and the respective block duration (the total is also indicated as mean ± SD, and range in parentheses).

Group	ID	GA (weeks)	Gender	Age (mo)	Weight (kg)	Block duration (s)			
						arm	leg	ipsi arm-leg	contra arm-leg
Full-term	F01	40	m	1.5	4.20	10	36	10	10
	F02	40	m	5	7.00	15	22	21	10
	F03	41	m	7.5	7.10	-	13	14	9
	F04	40	f	3	5.60	9	18	10	12
	F05	40	f	5.5	7.20	23	16	27	15
	F06	40	m	3	5.50	10	9	10	17
	F07	40	f	1.5	4.35	7	12	7	7
	F08	41	m	5	6.90	9	12	7	-
	F09	41	m	6.5	8.30	15	21	17	21
	F10	40	m	3	5.10	16	19	12	9
	F11	40	m	6	6.90	12	16	6	13
	F12	40	m	4	5.38	16	18	21	14
	F13	40	f	0.5	3.25	6	9	-	13
	F14	39	m	3	5.80	12	11	10	9
	F15	40	f	5	7.00	12	11	12	8
	F16	40	m	4	8.10	-	14	14	9
	F17	40	m	4	4.77	10	14	8	14
	F18	40	m	3	6.10	15	23	13	17
	F19	41	f	1.5	4.50	-	16	-	7
	F20	41	f	3.5	4.50	26	16	15	10
	Total			3.8 ± 1.4 (0.5–7.5)	5.8 ± 1.2 (3.2–8.3)	13.1 ± 4 (6–26)	16.3 ± 4.3 (9–36)	13 ± 4.2 (6–27)	11.8 ± 3.1 (7–21)
Preterm	P01	33	f	5.8	7.10	-	20	-	-
	P02	32	m	4	7.00	10/31	30/30	20/20	-
	P03	30	m	4	6.80	13	28	12	24
	P04	35	f	2	3.50	4	7	-	-
	P05	35	f	2	3.65	-	12	-	-
	P06	34	f	1.5	5.50	14	16	6	14
	P07	32	m	4	7.20	14	20	31	20
	P08	35	m	7	6.30	15	20	13	20
	P09	36	m	4	7.20	17	16	14	18
	P10	34	f	1	3.30	4	10	-	3
	P11	35	f	4.5	7.10	12	20	11	18
	P12	26	f	5	6.00	11	17	16	17
	P13	33	m	3	6.20	-	14	16	4
	P14	33	m	4	6.70	6	5	7	8
	P15	34	f	5.5	7.00	12	11	10	6
	P16	28	f	6	6.20	9	18	11	6
	P17	34	f	2.5	4.70	-	15/12	20	16
	P18	32	m	2	5.30	12	16/12	5/11	14
	Total			3.7 ± 1.3 (1–7)	5.9 ± 1 (3.3–7.2)	12.3 ± 4.1 (4–31)	16.6 ± 5.1 (5–30)	13.9 ± 4.9 (6–31)	13.4 ± 5.7 (3–24)

GA, gestational age. For child P02, P17, and P18, arm, leg, and ipsilateral arm-leg block conditions were recorded twice, but not consecutively (the duration of both blocks is indicated).

### Experimental setup and data recording

**Figure 1:**
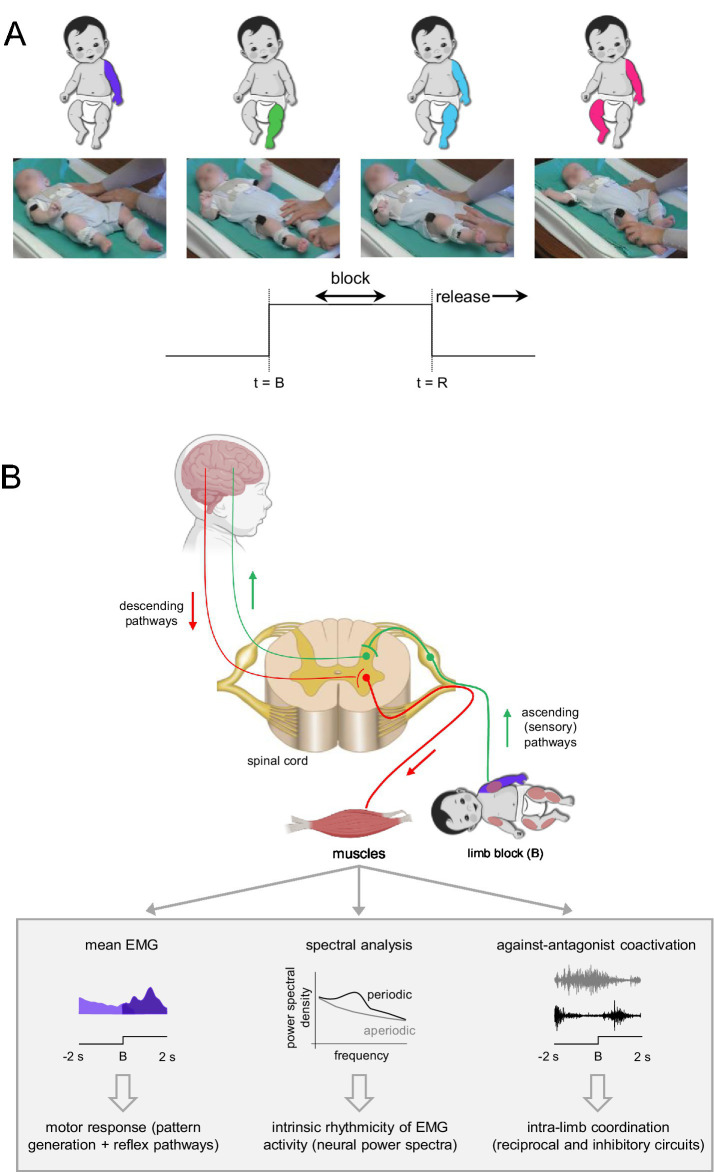
Experimental procedure and analyses. **(A)** Experimental setup for different limb block conditions in full-term and preterm infants: arm, leg, ipsilateral arm-leg, and contralateral arm-leg block. We analyzed the time intervals spanning 2 s before and after limb block (*t* = B) or release (*t* = R). **(B)** The schematic diagram illustrates the assessment of neural pathway functioning and sensorimotor responses to limb block during spontaneous limb movements. Three analytical approaches (lower panels) were used to evaluate EMG patterns following limb block (and release): changes in mean EMG activity, intrinsic rhythmicity of the neural drive (neural power spectra), and agonist–antagonist coactivation. These analyses were conducted within a 2-s pre- and post-block time window.

The experimental session lasted ~30 min (including placement of EMG electrodes). Before recording the different block limb conditions, infants were allowed to move freely during active wakefulness while lying supine on a standard medical couch for at least ~3–5 min. Thereafter, we proceeded to the execution of the four block conditions: arm, leg, ipsilateral arm-leg (block of arm and ipsilateral leg), and contralateral arm-leg (block of arm and contralateral leg) block (Figure 1A). During the experiments, all infants remained in the active and alert awake state; no sessions were recorded in which infants transitioned to quiet wakefulness or showed signs of drowsiness. Infants did not exhibit signs of distress (e.g., crying) during either spontaneous movements or limb block procedures. In most subjects, we also recorded spontaneous and passive movements in addition to limb blocks, and we have previously reported EMG activity characteristics during spontaneous and passive joint movements (Dolinskaya et al., 2023). Here we report the analysis of muscle activity during four different block limb conditions. Most infants had all block conditions recorded, and overall, we performed 17, 20, 18, and 19 recordings of arm, leg, ipsilateral arm-leg, and contralateral arm-leg blocks, respectively, for full-term infants, and 15, 21, 16, and 14 recordings for preterm infants (Table 1). The order of block limbs was randomized across infants. During the block, the limb was gently but firmly kept in a horizontal position (the leg extended below the hip and the arm positioned near the trunk, as shown in Figure 1A). Manual manipulations of flexion and extension joint movements are commonly used to assess muscle responses in infants in clinical settings (McIntyre et al., 2011; Marinelli et al., 2013). In our study, feasibility and infant comfort were prioritized by performing the limb block manually by the experimenter. Horizontal alignment and visual inspection of limb position during recording were used to maintain intra-rater consistency across infants and conditions. The limb block for the leg was executed slightly faster than that for the arm to achieve the horizontal position (since the leg was typically closer to the horizontal when the experimenter initiated the block). Nevertheless, the block of both upper and lower limbs was executed relatively quickly by the same experimenter (within ~0.3 s interval, and the end of this interval deemed the block’s onset). The duration of the block varied across the probes (3–36 s, Table 1), with a period of at least 4–5 s allowed between blocks.

EMG activity was recorded bilaterally using the Trigno Wireless System (Delsys Inc., bandwidth 20–450 Hz, overall gain 1,000, sampling rate 963 Hz) from the following muscles: biceps brachii (BB), triceps brachii (TB), rectus femoris (RF), biceps femoris (BF), tibialis anterior (TA), and gastrocnemius lateralis (LG). The size of the Trigno bar EMG electrodes was relatively small (5 mm) in order to minimize crosstalk. The skin was cleaned and gently rubbed with alcohol before the electrodes were placed. All movements were recorded by a digital video camera (PanasonicHC-V760EE-*κ*, 1920 × 1080pixels, 50 frames/s). The EMG and video recordings were synchronized.

### Data analysis

The recordings were first examined to determine the onset and release of limb blocks in each condition. Two independent experimenters examined the original video recordings and assessed the block intervals for each limb. In the majority of cases, there was significant consistency between observers: on average, the difference was ~1–2 frames of video recordings (~20–40 ms) pooling all limb blocks and infants together (and we used the mean between the two values as the onset). We compared EMGs recorded 2-s pre-block interval [−2 s, B] and 2-s post-block interval [B, 2 s] (where B is block onset). The rationale for analysing a specific time interval (2 s before and 2 s after block onset) was that if limb block is considered a disruption to continuing coordinated activity, it can be used as a probe to determine the presence of inter-limb coordination, similar to how limb block or loading during stepping (with cycle duration of ~2 s in infants) is used to examine automatic/reflexive responses (Lam et al., 2003; Pang et al., 2003; Dewolf et al., 2022). We also assessed whether there were changes during block release (comparing time intervals [−2 s, R] and [R, 2 s], where R is block release).

All data analyses were performed using custom-written programs in Matlab (MathWorks, Natick, MA). Initially, the raw EMG data were visually examined to identify artefacts and eliminate corrupted data segments (which were quite small: about 4%) from subsequent analysis. The EMG data were high-pass filtered at 60 Hz, notch filtered at 50 Hz, full-wave rectified and low-pass filtered at 3 Hz to obtain the envelope time series. All the filters were zero-lag fourth-order Butterworth filters. The EMG envelopes were time interpolated over a normalized 100-point time base. To evaluate the effect of limb block, we analysed changes in (1) mean muscle activity, (2) its spectral characteristics, and (3) antagonist coactivation (Figure 1B).

*Mean EMG activity.* To characterise the general features of muscle activity, we calculated and compared the mean amplitude of rectified EMG profiles before (−2 s, B) and after (B, 2 s) block onset. Nevertheless, it is important to note that measuring EMG amplitude in μV is a qualitative measurement due to variances in skin impedance across infants. In addition, while the 2-s analysis window was originally chosen to probe disruptions in ongoing coordinated activity (see above), we also examined whether varying the time window, using shorter (0.5 s) or longer (3 s) intervals, would influence the results.

*Spectral analysis.* In order to reveal changes in the frequency content of the EMGs of individual muscles, as it may occur in some limb movements (Dewolf et al., 2022), we applied the FOOOF algorithm (Donoghue et al., 2020; Sylos-Labini et al., 2022) that models the power spectral density (PSD) of EMG data as a combination of periodic and aperiodic components. The periodic activity *G_n_* is rhythmic, like neural oscillations, is identified by peaks in the power spectrum and is modelled as sum of N total Gaussians, described as:


(1) $$G_{n}\left(f\right)=A*\exp\left(\frac{-{\left(f-f_{c}\right)}^{2}}{2\sigma^{2}}\right)\;\;\;$$

where *A* is the power of the peak, *f_c_* is the central frequency, *σ* is the standard deviation (bandwidth) of the Gaussian, and *f* is the frequency vector.

Aperiodic activity *L* is non-rhythmic (e.g., white noise) and is modelled using a Lorentzian function, written as:


(2) $$L\left(f\right)=b-\log\left(k+f^{\chi }\right)\;\;\;$$

where *b* is the broadband offset, *χ* is the exponent, and *k* is the “knee” parameter, accounting for the bend in the aperiodic component. Broadband power refers to fluctuations occurring over a broad range of frequencies. The final outputs of the FOOOF algorithm are the parameters defining the best fit for the N Gaussians in Equation 1 and the aperiodic component in Equation 2.

*Correlation and coactivation of antagonist muscles.* Finally, we analysed potential changes in activation of antagonist muscles following the block. For analysing antagonist muscles, we calculated the Pearson correlation coefficient (*r*) and the coactivation index (*CI*) between pairs of antagonists (BB-TB, RF-BF, and TA-LG) in 2-s pre- and post-block intervals. The *CI* was assessed using the following formula (Rudolph et al., 2000; Mari et al., 2014; Martino et al., 2014; Dolinskaya et al., 2023):


(3) $$CI=\frac{\underset{N}{\sum }\left\{\frac{\left[EMG_{H}\left(j\right)+EMG_{L}\left(j\right)\right]}{2}\right\}*\left[\frac{EMG_{L}\left(j\right)}{EMG_{H}\left(j\right)}\right]}{N}\;\;\;$$

where *EMG_H_* and *EMG_L_* represent the antagonist muscle pairs’ highest and lowest activity, respectively, and *N* is the number of temporal points digitized in the selected interval ([−2 s, B] or [B, 2 s]). The *CI* was averaged over the entire interval duration (from 1 to *N*) in order to provide a global measure of the coactivity level. When this parameter is used Equation 3, high *CI* values indicate a high level of activation of both muscles, whereas low *CI* values indicate either a low-level activation in both muscles or a high-level activation in one muscle with a low-level activation in the other muscle in the pair.

### Statistics

Since the null hypothesis of normality of these data was rejected by performing the Shapiro–Wilk W-test (*p* < 0.05), we used non-parametric statistical methods. To compare independent samples, we used the Kruskal-Wallis test and the Mann–Whitney U test with a Holm-Bonferroni correction for the effects of full-term vs. preterm group, time interval before vs. after block onset, and block conditions on the EMG activity of six muscle pairs. In addition, for comparisons of mean EMG activity per subject and muscle, we calculated the rank-biserial correlation (rB) as a measure of effect size. The non-parametric Wilcoxon rank sum test was used to evaluate the differences between full-term and preterm infants in the average periodic and aperiodic parameters of PSD. The level of statistical significance was 0.05. All statistical analyses were performed using MATLAB software.

## Results

### EMG activity

**Figure 2:**
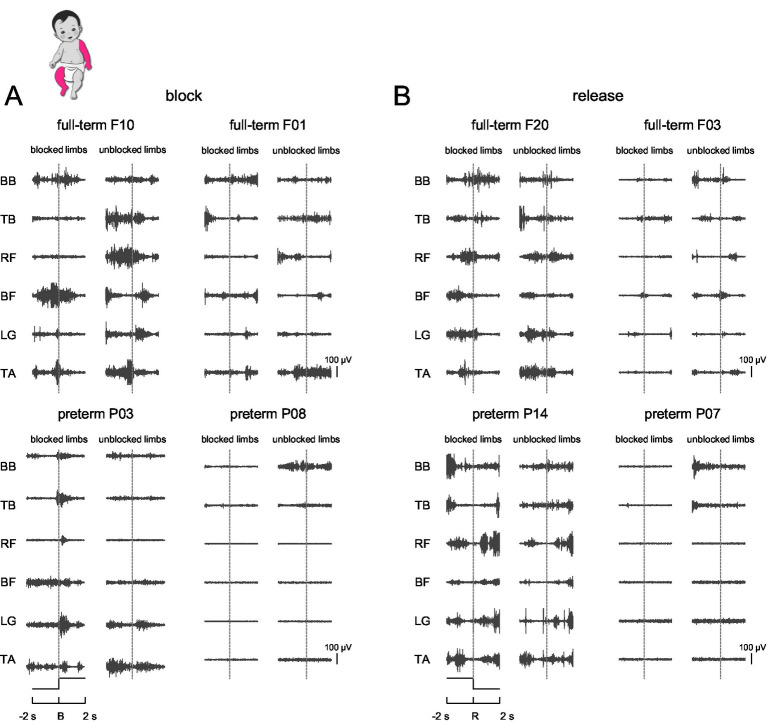
Examples of muscle responses to limb block. **(A)** Examples of EMGs for blocked and unblocked limbs in two full-term and two preterm infants during contralateral arm-leg block displayed for the time interval including 2 s before [−2 s, B] and after [B, 2 s] limb block. Vertical dotted lines indicate block onset (B, block). **(B)** Examples of EMGs for block release in four infants (R, release). Note variable EMG patterns during both limb blocks and releases. BB, biceps; TB, triceps; RF, rectus femoris; BF, biceps femoris; LG, gastrocnemius lateralis; TA, tibialis anterior.

Different limb block (arm, leg, ipsilateral arm-leg, contralateral arm-leg) conditions were tested in 20 healthy full-term and 18 preterm infants. Figure 2 shows examples of EMG traces during contralateral arm-leg block (panel A) and release (panel B) for full-term and preterm infants in the time interval including 2 s before and 2 s after limb block/release. The EMGs for each infant were separated into blocked and unblocked limbs. Before the block, the infant was allowed to move spontaneously in a supine position, so that some EMG activity could be detected before the block began, as well as after block release.

**Figure 3:**
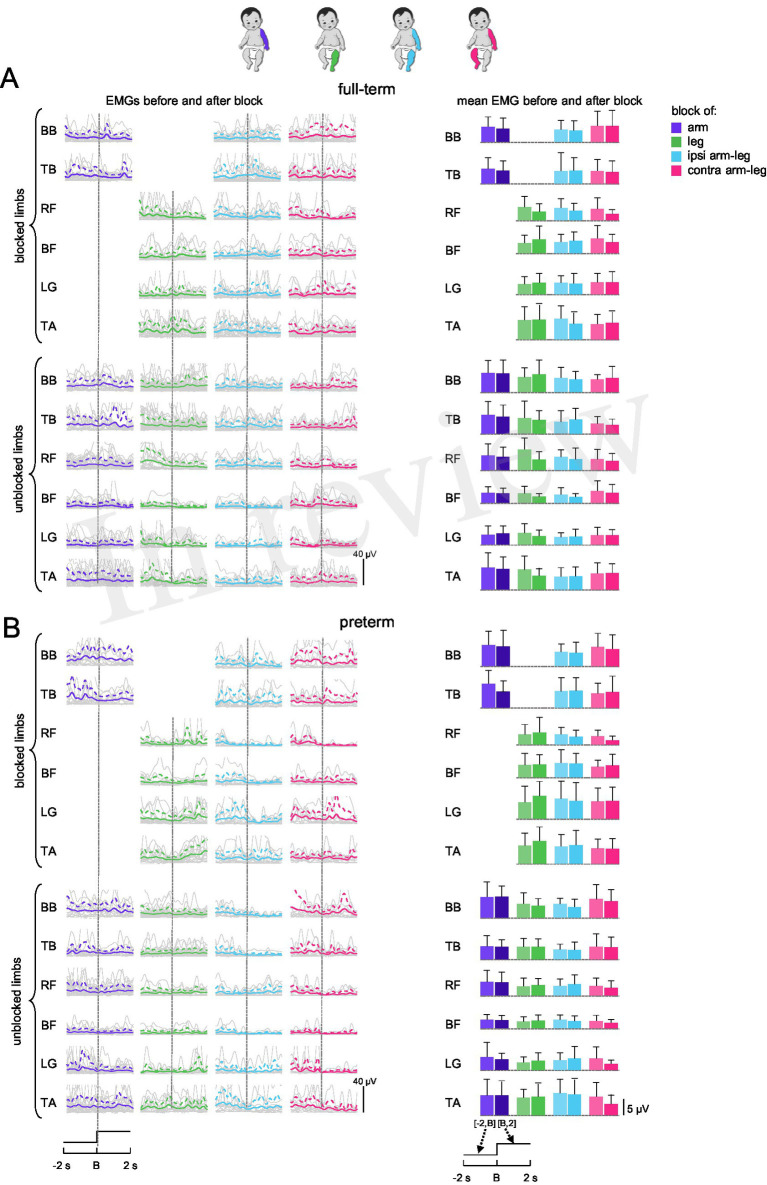
Muscle activity during limb blocks in full-term **(A)** and preterm **(B)** infants. Left panels: EMG data of blocked and unblocked limbs of individual subjects (in grey) and ensemble-averaged EMG profiles across subjects (in colour, mean + SD) during time interval [−2 s, 2 s] relative to block onset (*t* = B). Vertical dotted lines indicate block onset. Right panels: corresponding mean EMG activity (+SD) across subjects for each muscle and block condition. For single arm and leg blocks (two left columns), the EMGs of unblocked left and right limbs were pooled together. Note similar levels of EMGs before [−2 s, B] and after [B, 2 s] limb block.

Despite the standardized block condition performed, we did not find a stereotyped response but rather significant variability in time-varying EMG profiles among muscles, infants and groups, illustrated also in Figure 3 for all infants (left panels). We compared the mean levels of EMG activity before and after the block (Figure 3, right panels). Statistical tests showed no significant differences between time intervals (before and after block) for full-term and preterm infants and for blocked/unblocked muscles (*p* > 0.1, Kruskal-Wallis test and Mann–Whitney U post-hoc test). As a measure of effect size for these comparisons, we calculated the rank-biserial correlation (rB). Across muscles and conditions, rB values were generally small, ranging from approximately −0.48 to 0.28, consistent with negligible or weak effects. These results suggest that the EMG activity remained largely unchanged before and after the limb block across infants and muscles.

In addition to the variable (inconsistent) responses observed across infants (Figures 2, 3), we also did not find consistent muscle activation patterns within individual subjects across the four limb-block conditions (Figure 1A). This variability may reflect differences in responses between upper and lower limbs or could be related to the limited number of probes, as only one trial per block condition was available for most infants (Table 1). Nevertheless, these findings suggest that the transient effect of limb immobilization is neither evident nor consistent across limbs or participants (Figure 3).

**Figure 4:**
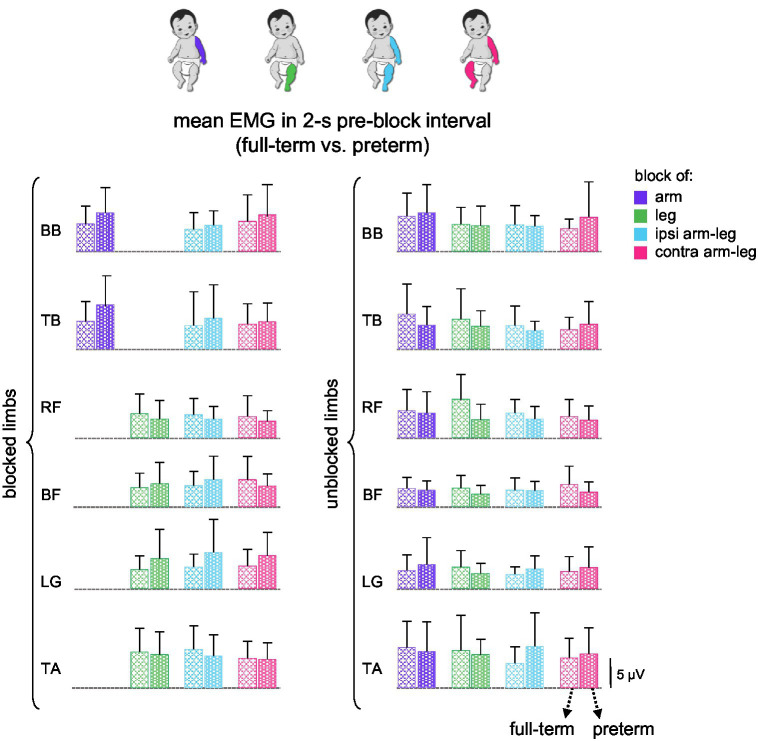
Mean muscle activity in full-term vs. preterm infants in the 2-s pre-block interval. Mean EMG activity (+SD) is shown (in μV) for both blocked and unblocked limbs. For single arm and leg blocks (two left columns), the EMGs of unblocked left and right limbs were pooled together.

Regarding group differences, we also assessed whether the general level of activity prior to the block differed between full-term and preterm infants. It is, however, worth noting that EMG amplitudes were evaluated in microvolts, which should be considered only a rough estimate of muscle activity. In most signal analysis protocols, comparisons are typically made relative to a baseline period representing resting activity. However, in the present study, defining a true baseline was not simple, as infants continuously performed spontaneous movements during active wakefulness and were rarely completely still. Therefore, we used the 2-s pre-block interval as the reference for ongoing motor activity before perturbation. Following this approach, we compared mean EMG values between full-term and preterm infants across all muscles and conditions in interval [−2 s, B] (Figure 4). No significant differences were found between groups (*p* > 0.03, Kruskal-Wallis test and Mann–Whitney U post-hoc test), consistent with the other comparisons reported.

Finally, the choice of the 2-s analysis window (2 s before and 2 s after block onset) was based on the rationale that the limb block transiently interrupts the ongoing coordinated motor activity, in a manner similar to how limb perturbations or loading during stepping (cycle duration ≈2–3 s in infants) are used to evaluate reflexive and adaptive motor responses (Lam et al., 2003; Pang et al., 2003; Dewolf et al., 2022). Nevertheless, we verified whether using somewhat different time windows, either shorter (0.5 s) or longer (3 s), would affect the results. For both shorter (0.5 s) or longer (3 s) window analyses, statistical tests showed no significant differences between time intervals (before and after block) for full-term and preterm infants and for blocked/unblocked muscles (*p* > 0.05, Kruskal-Wallis test and Mann–Whitney U post-hoc test).

### Spectral EMG analysis

**Figure 5:**
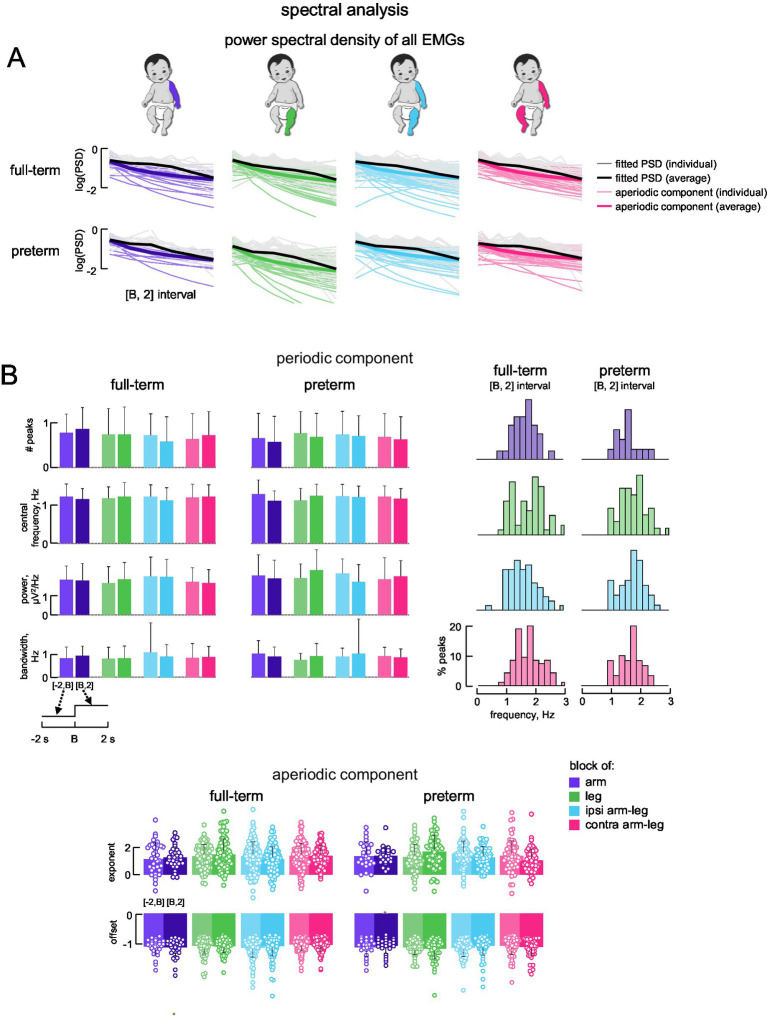
Parametrized frequency analysis of EMG data. **(A)** Power spectral density (PSD) for all full-term (upper) and all preterm (lower) infants in time interval [B, 2 s], calculated from rectified EMGs for all muscles together using Fast Fourier Transform (FFT) and fitted with an algorithm for parameterizing PSDs into periodic and aperiodic components. Grey lines represent fitted PSDs from individual subjects, black lines represent average fitted PSD across subjects, light coloured lines represent the aperiodic component from the individual PSDs, and dark coloured lines represent the average aperiodic component across subjects. **(B)** Characteristics of periodic and aperiodic components in the time intervals [−2 s, B] and [B, 2 s]. Upper panels: average (+SD) number of peaks, central frequency, power and bandwidth of the peaks across subjects and muscles are shown on the left; percent number of peaks for corresponding central frequencies are shown on the right. Lower panels: average (+SD) exponent (upper) and offset (lower) of the aperiodic component across subjects (the data for all muscles were pooled together). Circles denote the distribution of individual values (per subject and muscle) within the specified condition and time window.

To reveal the frequency content of the EMGs of individual muscles, we applied an algorithm (Donoghue et al., 2020; Sylos-Labini et al., 2022) that modelled the power spectral density (PSD) of EMGs as a combination of periodic and aperiodic components (Figure 5). Model fitting was accurate, with average *r*^2^ = 0.795 (95% confidence interval; 0.80 for full-term and 0.79 for preterm).

The periodic component of PSD for each muscle had no significant number of peaks: the average number of peaks for each condition and each time interval ([−2 s, B] and [B, 2 s]) resulted in <1 (all muscles pooled together) with no significant differences between groups (*p* > 0.05, Wilcoxon rank sum test; Figure 5B). Percent number of peaks for corresponding central frequencies are shown on the right of Figure 5B. The central frequencies of the peaks ranged from ~1–2.5 (~30% of peaks were in the range 1.9–2.3 Hz for both full-term and preterm infants), with a mean of ~1.2 Hz, and the percentage of central frequencies decreased toward 0 and 3 Hz (Figure 5B, right panels). The other parameters of the peaks in the spectra (peak central frequency ~1.2 Hz, bandwidth ~1 Hz, and peak power ~2 μV^2^/Hz) also showed no significant differences between block conditions, time intervals and groups (*p* > 0.05, Wilcoxon rank sum test; Figure 5B).

For the aperiodic component, the best model involved the knee parameter equal to 1 (k = 1) for all conditions (not shown), which was not different between the infant groups. The other aperiodic parameters, namely, the broadband offset and the exponent (*see Methods*), were also not significantly different between full-term and preterm infants (*p* > 0.05, Wilcoxon rank sum test; Figure 5B, lower panels).

### Correlation and coactivation of antagonist muscles

**Figure 6:**
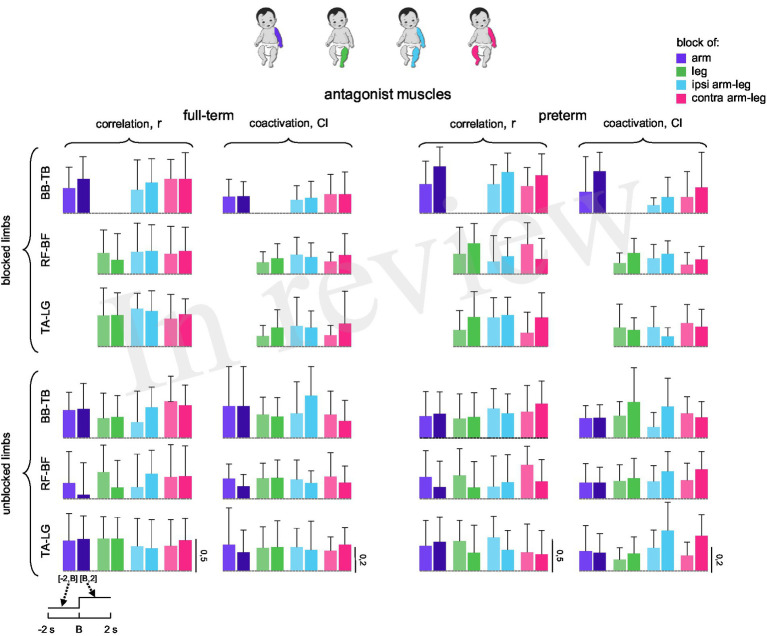
Correlation and coactivation of antagonist muscles during limb block in full-term and preterm infants. Correlation (*r*) and coactivation (*CI*) of antagonist muscles (BB-TB, RF-BF, and TA-LG) were calculated in the time intervals [−2 s, B] and [B, 2 s].

Coupling between pairs of antagonist muscles is often used to assess the relationship between their activity or changes in limb rigidity following perturbation (Rudolph et al., 2000; Mari et al., 2014; Solopova et al., 2019; Dolinskaya et al., 2023). Figure 6 illustrates the results of such analyses for antagonist muscles using parameters like Pearson’s correlation coefficient (*r*) and coactivation index (*CI*) before and after limb blocks. Overall, both *r* (~0.2–0.7) and *CI* (~0.1–0.3) were relatively small (Figure 6) and showed no significant difference between time intervals [−2 s, B] and [B, 2 s] for all block conditions and pairs of antagonist muscles (BB-TB, RF-BF, and TA-LG) for both full-term and preterm infants (*p* > 0.1, Kruskal-Wallis test and Mann–Whitney U post-hoc test).

### Changes in muscle activity during limb block release

We also examined potential changes in EMG activity during limb block release, comparing the 2-s pre- and post-release intervals. Similar to the onset of limb block (Figures 3, 5, 6), we did not find differences in the levels of EMGs before [−2 s, R] and after [R, 2 s] limb release (not shown) (*p* > 0.05, Kruskal-Wallis test and Mann–Whitney U post-hoc test), and we observed significant variability in time-varying EMG profiles (Figure 2B), as during limb block. Also, the periodic and aperiodic spectral EMG components (*p* > 0.05, Wilcoxon rank sum test) and agonist–antagonist muscle coactivation (*p* > 0.1, Mann–Whitney U post-hoc test) were not different in 2-s pre- and post-release intervals.

### Effect of age on muscle activity following block

While we were interested in the overall effect of limb block during spontaneous movements during the first 7 months after birth (Figures 3–6) [SMs are typically observed within 5–7 months after birth (Jensen et al., 1995; Dolinskaya et al., 2021)], the kinematics of SMs show some age-related changes (Prechtl and Hopkins, 1986; Piek and Carman, 1994; Gima et al., 2011; Kanemaru et al., 2012; Ohmura et al., 2016). Therefore, we also verified whether there were systematic age-related differences in the EMG activity of a given upper or lower limb muscle following its block (pooling all conditions when the corresponding limb was blocked or not). To this end, infants (Table 1) were separated into three age groups (<2.5 mo, 2.5–4.5 mo, and 4.5–7.5 mo). We found no significant age-related differences in mean EMG activity before and after block for any muscle or age group (*p* > 0.05, Kruskal-Wallis test).

## Discussion

We examined whether infants (both full-term and preterm) during the first half-year after birth reveal changes in how muscles respond to the temporary mechanical block of one or two limbs during SMs, while the other limbs were allowed to move. To this aim, we analysed polymyographic recordings in the lower and upper limb muscles using multiple analytical approaches, including time-domain EMG averaging, power spectral density analysis, and coactivation/correlation indices (Figure 1B, 3–6). Infants did not appear to discontinue their ongoing muscular activity immediately after the block, whether in the blocked or unblocked limbs, which was still noticeable (Figure 3), albeit varying between limbs and infants (Figure 2). While the analyses (Figures 3–6) only represent an ‘integrative’ measure of coordination, across all of them no consistent or stereotyped EMG responses were found in either the blocked or the unblocked limbs during the 2-s pre- and post-block intervals.

### Interpretation of the lack of consistent responses

Mechanical stimulations are often employed to assess responsiveness or inter-limb sensorimotor connections throughout early development, but the results reported in the literature are diverse, depending on the experimental paradigm and developmental stage investigated.

For instance, kicking supine with a load at the ankle may make either the duration of flexion or extension of the loaded leg longer (Musselman and Yang, 2007) although the authors did not find significant changes in the muscle activation. Some previous studies reported changes in the interlimb coordination in human infants in response to unilateral weighting of the limb, that changes the proprioceptive input and/or limits limb movements. Infants may respond to the unilateral weight during SM with an increase in kicks of the non-weighted leg compared to the number of kicks in the weighted leg (Thelen et al., 1987; Vaal et al., 2000), consistent with studies on newborn animals (Brumley and Robinson, 2013), suggesting a role of proprioception and movement-related feedback to modulate spontaneous motor activity during early motor development. Limb weighting experiments used paradigms in which kicking behaviour (from one to several minutes) was evaluated during a baseline period and again when the weight was added to one of the limbs. However, for such proprioceptive manipulations, infants typically began to change their pattern of leg kicking after some period of exposure to unilateral weighting or an interlimb yoke (Thelen, 1994) in line with a delayed onset of such adjustments in perinatal rats (Robinson et al., 2008), implying a general non-specific effect rather than an automatic/reflexive response, whereas muscular responses to limb block may differ in functional role and underlying mechanisms.

To our knowledge, there has been no comprehensive investigation of alterations in the coordination of activity across different muscle groups in response to limb block during SMs. Given that inter-limb coupling during early spontaneous activity is known to be weak but significant, both in kinematic correlations between upper and lower limbs during stepping (La Scaleia et al., 2018; Forma et al., 2019) and during spontaneous movements (Piek et al., 2002; Kanemaru et al., 2012), we also employed a two-limb block (ipsilateral and contralateral, Figure 1A), to test whether proprioceptive feedback from multiple limbs might enhance such coupling. Averaged muscle activity (Figures 3, 4), its spectral characteristics (Figure 5), and against-antagonist coactivation (Figure 6) were similar before and after the block of different limbs in both full-term and preterm infants. The results (Figures 3–6) corroborate generally poor velocity and position correlations of the limb endpoints during SMs in healthy infants. For example, the pattern of spontaneous movements changes from a general activity involving all limbs to an activity involving more selective interlimb coordination from 2 to 4 months of age; however, such correlations are significant but weak, requiring a large number of movements to reveal them (Kanemaru et al., 2012). Therefore, if one considers the applied manipulation (limb block) as a disruption to continuing coordinated muscle activity, the lack of consistent responses (Figures 3, 5, 6) may be interpreted as a manifestation of immature and weakly integrated sensorimotor connections between the limbs during SMs.

The lack of consistent responses during limb block is opposed to the rapid and coordinated responses observed during stepping movements or manipulations related to muscle tone examination in similar age ranges. For example, applying load of one leg or trip-inducing stimuli during stepping movements can elicit immediate responses, such as an increase in hip and knee flexor muscle torque (Lam et al., 2003; Pang et al., 2003), and transient leg block and release may evoke immediate tonic or phasic muscle reactions (Dewolf et al., 2022). Other manual manipulations, such as passive joint flexions and extensions, are able to reveal consistent muscle reactions to lengthening and shortening, which is a powerful manifestation of developing muscle tone in infants, and it is quite impressive that such reactions in some muscles occur in a high proportion (>90%) of passive movements (Solopova et al., 2019). Thus, the presence of sensory input during changes in muscle length is likely to be more effective in revealing sensorimotor connections throughout early development than its non-appearance (during limb block).

### Neurodevelopmental and clinical implications

The absence of systematic EMG changes likely reflects a genuine physiological characteristic of early motor organization. During the first months after birth, neural circuits supporting inter-limb coordination are functionally present but still immature. Spinal networks can generate segmental reflexes and basic motor patterns, while supraspinal inputs are emerging and only weakly integrated with spinal circuits (Yang et al., 2015; Blumberg and Adolph, 2023). Coupling between limbs depends on the gradual maturation of sensorimotor feedback loops. In this context, a transient limb block during SMs may not yet trigger organized responses, as the developing system is not fully tuned to integrate proprioceptive signals across limbs. This interpretation is consistent with developmental neurophysiology: although muscle tone and segmental reflexes can be elicited early, the integration of sensory feedback into coordinated inter-limb responses emerges gradually through experience-dependent plasticity (Schouenborg, 2010; Hadders-Algra, 2014).

The sample of preterm infants (Table 1) was heavily biased toward moderate and late preterm infants (overall, 15 out of 18 preterm infants had 32 or more weeks of gestational age). Therefore, we do not know whether a sample more representative of extreme and very preterm infants would display behaviour comparable to that reported here. This point is relevant also with regard to potential clinical implications, since it is well known that infants at risk of developing cerebral palsy or other neurodevelopmental motor disorders mainly fall in the categories of extreme and very preterm infants, i.e., they are born before 32 weeks of gestational age and/or weigh less than 2.5 kg at birth. Clinically, the absence of systematic adjustments, as observed in our healthy sample, may be used as a baseline reference for identifying early deviations in motor coordination, potentially informing early assessment and intervention strategies. Variability is an inherent property of sensorimotor behaviour at any age of life (Vidal and Lacquaniti, 2021). As far as it concerns early development, the presence of consistent or exaggerated responses to brief limb manipulations could serve as an early indicator of atypical sensorimotor development, such as hyperconnected pathways or altered inter-limb coupling. For instance, a well-established feature of motor behaviour in infants with cerebral palsy is the stereotyped nature of their SMs, which contrasts with the variability and flexibility observed in typically developing infants (Hadders-Algra, 2018; Kwong et al., 2018).

### Limitations

The study has some limitations. First, standardizing the exact moment of limb block was challenging due to the inherent variability of spontaneous movements. A larger number of trials might be necessary to detect relationships between transient limb block and the phase or type of SMs episodes (e.g., ‘writhing’ or ‘fidgety’, Prechtl and Hopkins, 1986). Second, the sex distribution of our sample was unequal (full-term: 7 females and 13 males; preterm: 10 females and 8 males). Although not inherently problematic, sex-related differences in motor development have been reported (Prechtl and Hopkins, 1986; Dinkel and Snyder, 2020). However, these differences also appear to be influenced by a combination of biological and socio-cultural factors, which are difficult to fully control in this type of protocol. Third, our analysis treats each group (full-term and preterm) as homogeneous, although some infants may have shown a response while others did not, depending on factors such as age or neurological maturity. More consistent responses might therefore be observed in certain individuals, but a larger number of probes would be required to examine interindividual variability, particularly in the context of identifying early indicators of atypical sensorimotor development. Nevertheless, when considering the full populations of full-term and preterm infants, the findings indicate that the transient effect of limb immobilization is neither evident nor consistent across limbs or participants (Figures 3, 5, 6). Despite these limitations, the use of three complementary analytical approaches (amplitude, spectral, and coactivation analyses; Figure 1B) strengthens confidence that this null finding does not reflect methodological artefacts but rather reflects immaturity of interlimb coupling.

## Conclusion

In sum, the results suggest that the lack of consistent muscle responses to limb block (Figures 3–5) may be indicative of weak inter-limb coordination during SMs, as compared to similar manipulations during stepping movements (Lam et al., 2003; Dewolf et al., 2022). Infant motor activity during the first half-year after birth is essential for the development of voluntary motor skills, muscle tone, and calibration of the proprioceptive system. High variability of neuromuscular signals and responses during SMs may attest neonatal immaturity, but they also involve potential benefits for learning locomotor tasks (Prechtl and Hopkins, 1986; Schouenborg, 2010; Sylos-Labini et al., 2022; Dolinskaya et al., 2023). Further research may provide new empirical evidence on the emergence of muscle coordinative behaviour, which has clinical implications for abnormal development (Hadders-Algra, 2014; Kanemaru et al., 2014) as well as the mechanisms driving early maturation of sensory circuitries.

## Data Availability

The raw data supporting the conclusions of this article will be made available by the authors, upon reasonable request.
